# A Rare Malposition of a Left Internal Jugular Central Venous Catheter into the Left Internal Mammary Vein

**DOI:** 10.5811/cpcem.2022.12.58202

**Published:** 2023-01-24

**Authors:** Christian A. Koziatek, Damilola Idowu, Richard White

**Affiliations:** * NYU School of Medicine, Ronald O. Perelman Department of Emergency Medicine, New York, New York; † Bellevue Hospital Center, Department of Emergency Medicine, New York, New York

**Keywords:** central venous catheter, internal mammary vein, internal jugular vein, central access, case report

## Abstract

**Case Presentation:**

We describe a case of left internal jugular central venous access with rare malpositioning into the internal mammary vein. Despite various confirmatory measures at the time of placement including ultrasonography of the internal jugular vein, as well as blood gas analysis consistent with venous blood by oxygen saturation and good venous flow in all three ports of the catheter, subsequent imaging confirmed misplacement into the internal mammary vein.

**Discussion:**

Central venous access is a frequently used procedure by emergency physicians for a variety of indications. Emergency physicians must be facile with both the technical process of central venous catheter placement, as well as possible pitfalls and complications of the procedure. Common complications, such as bleeding, pneumothorax, arterial injury, infection, and hematomas, are usually well known; less frequently encountered is malposition of the catheter despite seemingly appropriate placement.

## CASE PRESENTATION

A 39-year-old female patient with a known history of intravenous (IV) drug use presented to the emergency department with fever, alteration of mental status, tachycardia, and hypotension. Intravenous access was difficult, and only a small-bore peripheral venous line could be obtained. Due to persistent hypotension, the decision was made to place a central venous catheter for reliable IV access and to initiate vasopressor therapy. A multilumen central venous catheter was placed under ultrasound guidance by Seldinger technique after left internal jugular vein puncture. The J-tip guidewire was advanced without resistance to a depth of about 20 centimeters (cm); the skin was dilated and the catheter was advanced over the wire without resistance. The catheter was fixed at 16 cm depth. After placement, ultrasonography of the neck confirmed the presence of the catheter in the internal jugular vein; all three ports flushed and aspirated blood easily. A venous blood gas sample was sent to the laboratory and the oxygen saturation (40%) was consistent with venous placement.

**Image 1:**
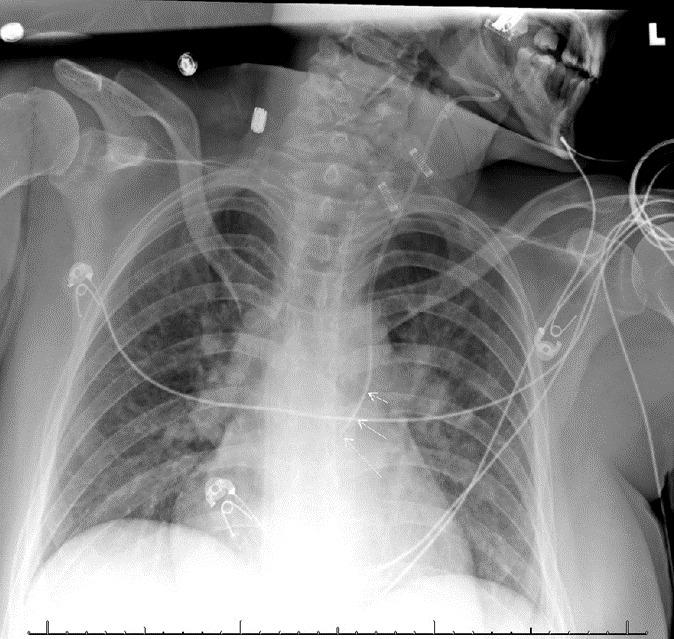
Anterior-posterior chest radiograph demonstrating the central venous catheter coursing atypically, over the left mediastinum, not crossing the midline (arrows).

**Image 2:**
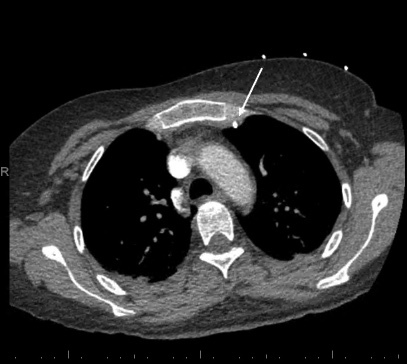
Coronal slice of chest computed tomography demonstrates the central venous catheter within the internal mammary vein, to the left and posterior to the sternum (white arrow).

**Image 3:**
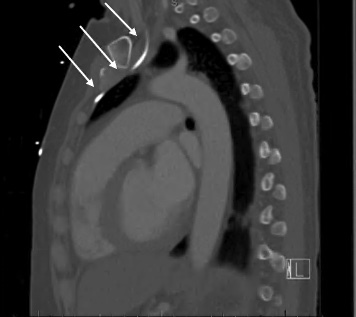
Saggital slice of chest computed tomography demonstrates the central venous catheter within the internal mammary vein, posterior to the sternum, coursing anterior to the great vessels and pleural space (white arrows). Volume 7, no. 1: February 2023 53 Clinical Practice and Cases in Emergency Medicine

Anterior-posterior chest radiograph (CXR) obtained after placement of the central venous catheter revealed the catheter taking an atypical course, projecting over the left mediastinum, not crossing the midline (Image 1). A computed tomography of the chest demonstrated the catheter coursing from the left internal jugular into the left internal mammary vein (Images 2 and 3). The patient was ultimately admitted to the intensive care unit, and the central venous line was removed at the bedside and replaced.

## DISCUSSION

We describe an unusual malpositioning of a central venous catheter into the internal mammary vein. Some complications of central venous access are well chronicled;^1^ however, internal mammary vein cannulation is a known but rarely reported complication, with only a handful of cases in the literature.^2^–^5^ Although a number of more common complications can be quickly ruled out without imaging – blood gas analysis and ultrasound imaging at bedside, for example, can confirm venous system placement and rule out pneumothorax^1^ – in this case, only on CXR was the misplacement identified. Chest pain during placement and/or aspiration/flushing has been reported as a possible sign of this specific malposition site, 4 but in an altered or critically ill patient, this may not be a reliable indicator, as in this case.

*CPC-EM Capsule*

What do we already know about this clinical entity?

*Some complications of central venous catheter placement are more common and well known, including bleeding, pneumothorax, arterial injury, infection, and hematomas.*

What is the major impact of the image(s)?

*This case describes a rare malpositioning complication into the internal mammary vein despite confirmatory measures that suggested good placement.*

How might this improve emergency medicine practice?

*Emergency physicians should remain vigilant for these rare complications and always confirm placement in multiple ways to assure appropriate catheter placement.*

Chest radiography will demonstrate a catheter coursing over the left side of the chest, which can also be seen in other misplaced central venous lines (pleural space, arterial, etc) While misplacement into arterial, soft tissue, or other non-venous sites is usually quickly identified via the above confirmatory measures, misplacement into unusual, undesired venous sites, which could also include the pericardiophrenic vein or the subclavian vein, may only be recognized with subtle abnormal imaging findings. A misplaced catheter in this position should not be used due to the risk of vessel damage and should be removed. Emergency physicians should be aware of the rarer possible complications of this commonly performed procedure and their appearance on imaging studies.
